# AutoML based workflow for design of experiments (DOE) selection and benchmarking data acquisition strategies with simulation models

**DOI:** 10.1038/s41598-024-83581-3

**Published:** 2024-12-31

**Authors:** Xukuan Xu, Donghui Li, Jinghou Bi, Michael Moeckel

**Affiliations:** 1 Aschaffenburg University of Applied Sciences, Faculty of Engineering, Aschaffenburg, 63743 Germany; 2 Dresden University of Technology DE, Faculty of Engineering, Dresden, 01069 Germany

**Keywords:** Mechanical engineering, Computational science

## Abstract

Design of experiments (DOE) is an established method to allocate resources for efficient parameter space exploration. Model based active learning (AL) data sampling strategies have shown potential for further optimization. This paper introduces a workflow for conducting DOE comparative studies using automated machine learning. Based on a practical definition of model complexity in the context of machine learning, the interplay of systematic data generation and model performance is examined considering various sources of uncertainty: this includes uncertainties caused by stochastic sampling strategies, imprecise data, suboptimal modeling, and model evaluation. Results obtained from electrical circuit models with varying complexity show that not all AL sampling strategies outperform conventional DOE strategies, depending on the available data volume, the complexity of the dataset, and data uncertainties. Trade-offs in resource allocation strategies, in particular between identical replication of data points for statistical noise reduction and broad sampling for maximum parameter space exploration, and their impact on subsequent machine learning analysis are systematically investigated. Results indicate that replication oriented strategies should not be dismissed but may prove advantageous for cases with non-negligible noise impact and intermediate resource availability. The provided workflow can be used to simulate practical experimental conditions for DOE testing and DOE selection.

## Introduction

The Design of Experiments (DOE) is a systematic method to determine the relationship between factors affecting a predefined target parameter. It is utilized to optimize processes, improve quality, and identify critical factors^1^. In recent years, the development of Cyber-Physical Systems (CPS) has significantly expanded the sources of data^2^. The application of Machine learning (ML) techniques for data analysis has rapidly spread in the industry^3^. However, DOE strategies based on statistical planning face challenges in coping with the exponential growth of data amount as well as dimensions. For instance, a three-level Full Factorial Design (FFD) for ten factors requires $3^{10}$ data points. The cost and effort associated with data collection in such scenarios are often prohibitive for typical engineering applications. In response, classical DOE provides solutions such as fractional factorial design, where only a fraction of the treatment combinations are sampled. Moreover, the space-filling design on computer simulation spreads out data points with the aim of maximizing the diversity of data points. The spread of training samples enables smooth interpolation, leading to accurate prediction of the trained models for further responses^4^. Apart from these model-free strategies, model-based DOE offers another perspective on data point sampling: choosing to spread out data points while observing the yield target parameter. A regression model is then fitted in a supervised manner, which will guide the data points sampling sequentially in one-shot or batch design. According to G. Franceschini, AL DOE can be considered as a subfield of model-based DOE^5^. Our work regarding model-based DOE is limited to the AL perspective. In contrast to model-free sampling strategies, the AL sampling is a series of model-driven sampling strategies^6^. By utilizing initial data resources to develop a predictive model for the target parameter, areas that are most in demand for exploration within the remaining parameter space can be identified through uncertainty or information entropy quantified from the predictive model^7,8^. Focused data sampling in these specific areas can efficiently reduce model prediction errors. Such intelligent sampling strategies maximize the utilization of limited data resources, thus improving model learning efficiency and accuracy^5^.

The compatibility of classical DOE strategies with ML-based data analysis has been explored. The implications of ML for DOE in quality management have been assessed by J. Freiesleben et al. from the perspective of ’ML for DOE’^9^. R. Arborett et al. systematic reviewed the joint application of DOE and ML in industrial production for product innovation^3^. Another of her studies discusses the selection of experimental designs for a given amount of data in conjunction with ML model performance, aiming to identify optimal combinations of designs and ML models for data collection and analysis^10^.

Although various AL sampling strategies have demonstrated superiority in respective studies, the lack of open-source code impedes the further research and validation of these strategies. Of greater concern is the fact that these AL sampling strategies are often executed by selecting the next data points from pool datasets (benchmarking datasets) split from a pre-generated raw dataset rather than sampling data according to the actual DOE logic^11,12^. This deviation raises concerns about the practical applicability of AL sampling in real-world scenarios.

This work proposes a robust workflow for conducting a comparative study of DOE strategies using Automated machine learning (AutoML)^13^. How to pick the appropriate DOE for a practical application scenario for carrying out experiments or production is the question this work seeks to answer.The objective of the DOE strategy in this study is limited to exploring the given parameter space as much as possible in order to obtain a mathematical model for predicting further responses under the constraint of predefined data resources. Obstacles affecting the fair evaluation of DOE strategies, such as suboptimal modeling, evaluation uncertainty of the models, and the inherent uncertainty in AL sampling strategies, are quantified and discussed. The paper is organized as follows: “Methods” section systematically elucidates the AutoML-based DOE comparative workflow. It discusses the potential uncertainties encountered during the evaluation of DOE strategies and proposes methods to mitigate these effects. A DOE comparative study is conducted in the absence of noise contamination with Central Composite Design (CCD), a Latin Hypercube Design (LHD), and three AL sampling strategies as candidates. Comparation between different DOEs as well as data allocation with or without replication are conducted in scenarios with noise on target parameter.The performance of DOE strategies under data uncertainty are documented and discussed in this work. Finally, guidance is provided on applying the proposed workflow to DOE testing and DOE selection.

## Methods

The AutoML-based workflow for conducting DOE comparative experiments is explained in detail in this Section. Additionally, it addresses AL sampling strategies, focusing on a reproduced committee strategy and a data sampling strategy using Monte Carlo Dropout for uncertainty assessment.

### AutoML-based workflow for DOE comparison studies

**Fig. 1:**
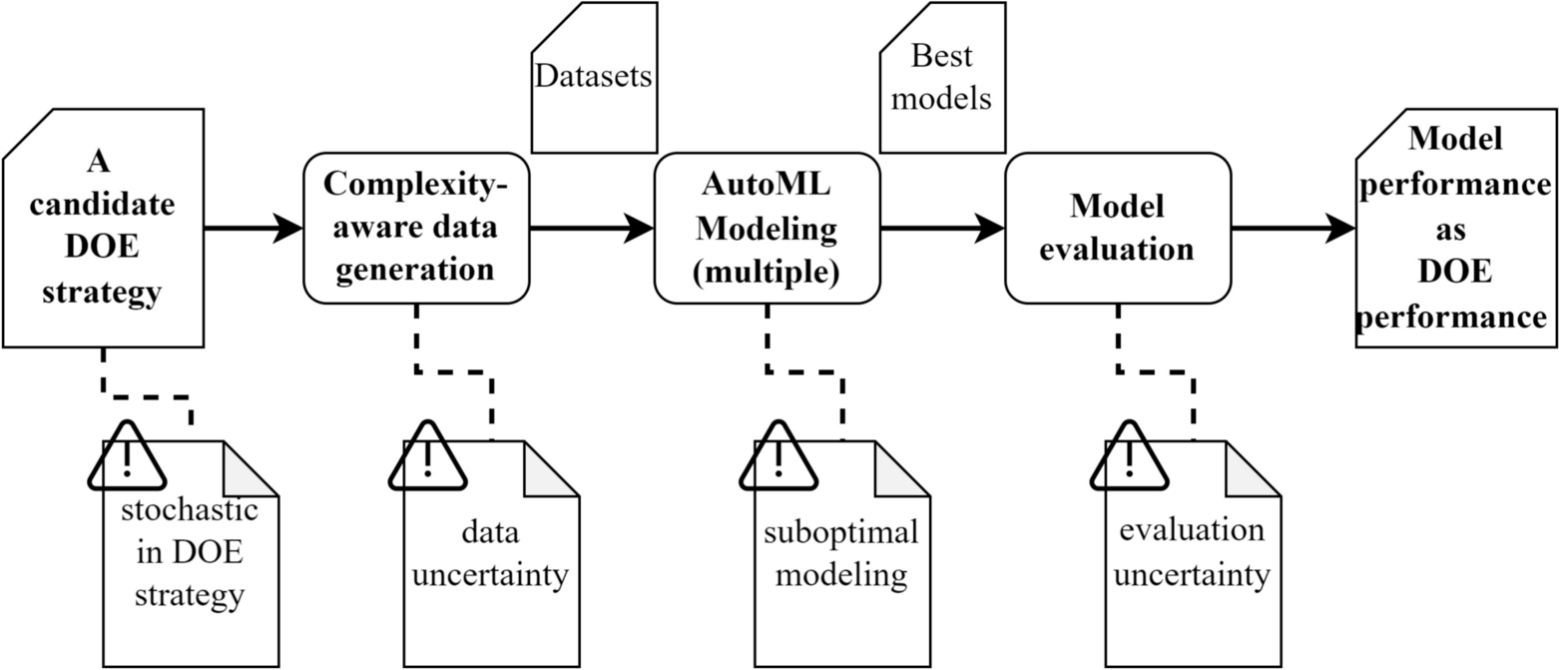
AutoML-based workflow for evaluating the performance of a chosen DOE strategy, possible impediments at each step are tagged.

The workflow diagram for a DOE comparison study using AutoML is summarized in Fig. 1.

The core of this workflow as shown in Fig. 1, is to quantify the superiority of a DOE strategy in terms of the performance of an optimal predictive model trained on the dataset generated according to this DOE strategy. First, selected DOE strategies guide the data generation (in the case of simulation experiments) or the data collection to construct corresponding datasets for modeling. Since one of the goals of DOE is to minimize the number of experiments required to analyze a given number of parameters, deleting some of the data points may disrupt the expected structure of the data distribution, causing missing information for parts of the parameter space that leads to the development of poorly performing or even incomplete models^14^. This phenomenon is even more prominent in small-data scenarios^15^ where the total amount of data is scarce. Thus, in contrast to the conventional splitting of the original dataset into training and test sets, the datasets generated by DOE strategies are employed purely as training sets for modeling. Accordingly, test sets containing a large number of data points need to be constructed for model evaluation.

AutoML aims to automate the construction and optimization of machine learning models. It automates processes such as hyperparameter tuning and feature selection, rapidly testing various algorithms and parameter combinations to identify the optimal modeling strategy^16^. In the comparative study of DOE, auto-sklearn is employed to perform the modeling tasks^17^. For each generated dataset, modeling is independently conducted multiple times. As an end-to-end model development pipeline, AutoML ensures that all modeling tasks are performed under a unified framework in addition to the rapid completion of repetitive modeling tasks^18^.The constructed models are then tested on a separately collected test set, which consists of a large number of data points. This ensures a fair assessment of the model performance with minimal evaluation uncertainty and modelling deviations. The performance of the optimal model obtained from the training set is regarded as the performance of the corresponding DOE strategy that guided the collection of the training set. Within this workflow, several factors-such as the inherent stochasticity of some DOE strategies, noisy data, suboptimal model training, and evaluation uncertainty, can impede the pursuit of accurate estimation of the DOE strategies.

#### Complexity-aware data generation

For DOE comparative studies, it is essential to evaluate different DOE strategies across various parameter spaces, as the performance of the same DOE strategy varies depending on the parameter space. In this study, a practically motivated concept of complexity is used to distinguish between different parameter spaces. Complexity is not solely determined by the number of input parameters in the parameter space. A parameter space of higher dimension is not invariably more complex than a lower dimensional one. The mathematical relationship between input and target parameters is the primary determinant of complexity. In this work, complexity is quantitatively defined as the data volume required to train a surrogate predictive model with AutoML to achieve a minimum performance of 0.9 R squared (R$^{2}$) score on a pre-constructed large test set.

Statistically planned DOE strategies are inherently deterministic. Once the parameter space (including the factors and their ranges) is defined, the locations of the planned data points are then derived by the DOE strategy. In contrast, other DOE strategies, such as LHD and AL sampling strategies, exhibit dependencies on input data, model (hyper-)parameters or random number initializations. For LHD, this stochasticity primarily manifests in the specific locations of sample points and their combinations across dimensions^19^ . This uncertainty can be controlled by fixing random seeds in Python. The stochasticity in model-based AL sampling strategies stems from their core predictive models. When the predictive models are built on initial datasets, variations in hyperparameters and settings of training parameters may result in different training outcomes, the planned positions of data points derived from computed uncertainties may differ. However, by fixing all random seeds and clearly defining all training parameters, such stochasticity can be controlled.

#### Stochastic sampling in DOE strategies

**Fig. 2:**
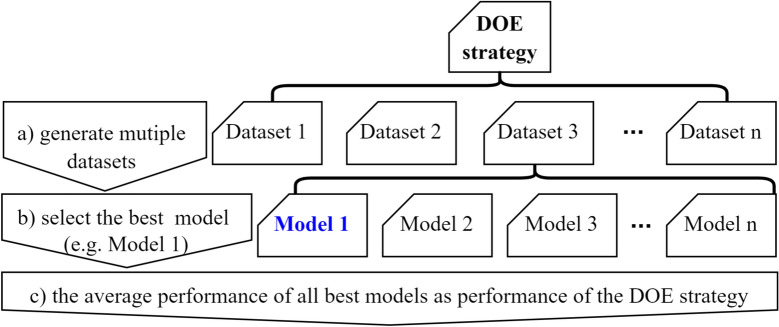
Assessment of DOE strategies requires multiple datasets and multiple AutoML runs, the performance of a DOE strategy is defined as the average performance of the best models.

To fairly evaluate the performance of DOE strategies with stochastic sampling, multiple datasets need to be generated. As illustrated in Fig. 2, for each generated dataset, the best performing model (assumed to be Model 1 in the figure) is identified as the performance of this DOE strategy on this dataset. The average performance of the optimal models obtained on these datasets is considered as the performance of the corresponding DOE strategy.

#### Uncertainty in data

Uncertain data interfere with data analysis: uncertainties unavoidable in data collection, e.g. limitations in calibration and sensor precision; systematic and stochastic uncertainties, e.g. unknown variables of interest or fluctuations in parameters cannot be controlled entirely. To compare the performance of different DOE strategies in data collection involving noise, multiple datasets containing noise need to be collected. The average performance of the optimal models obtained on these datasets is considered as the performance of the DOE strategy for a specific noise contamination. This uncertainty arising from inaccurate data couples with the uncertainty of the DOE strategy. Consequently, it may require more effort for dataset generation to obtain a fair assessment of the DOE strategy. In this article, we conduct 2 DOE comparison studies in the presence of noise contamination, taking noise characterized by uniform distribution as an example.

#### Suboptimal modelling

AutoML modeling in comparative DOE studies aims to achieve the best possible model performance on the given dataset. Models that deviate from the optimal hyperparameter settings perform worse than the optimal model, introducing a systematic bias between the performance of the model and the performance of the corresponding DOE strategy. To minimize such bias as much as possible, multiple AutoML modeling tasks are necessary in pursuit of optimal modeling. During modeling, AutoML modeling tasks are carried out multiple times, while the model with the highest R$^{2}$ among all trained models is considered indicative of the performance of the DOE strategy.

#### Uncertainty in model evaluation

Claudia Beleites et al. highlights the significant uncertainty in evaluating model performance with limited test data^20^. This uncertainty can obscure the real performance gap between the two models. To ensure a fair assessment of the trained models from AutoML, the test set must include a sufficient amount of data. For a test set containing *n* data points, the confidence interval for model evaluation can be calculated.

1 $$\begin{aligned} {CI} = \bar{x} \pm t \left( \frac{s}{\sqrt{n}} \right) \end{aligned}$$

The *t* in equation (1) can be obtained by consulting a T-distribution table. Note that the t-statistic is not directly applied to the training data (which may contain noise contamination). Instead, we apply the t-statistic to the error between model predictions and the true value of the target parameter within test data set to assess the uncertainty of the model performance. We randomly selected some models and determined that this distribution follows a Gaussian distribution. For *n* greater than 1000 *t* can be set to 1.96. Using R$^{2}$ score as the metric, a regression model can be evaluated on a test set containing 200,000 data points with a precision of better than ±0.002 (95 % confidence interval). The standard deviation *s* of observed performance can be considered less than 0.5, given that the R$^{2}$ score of a trained model typically ranges between 0 and 1.

### Complexity-aware data generation with impedance simulation

**Fig. 3:**
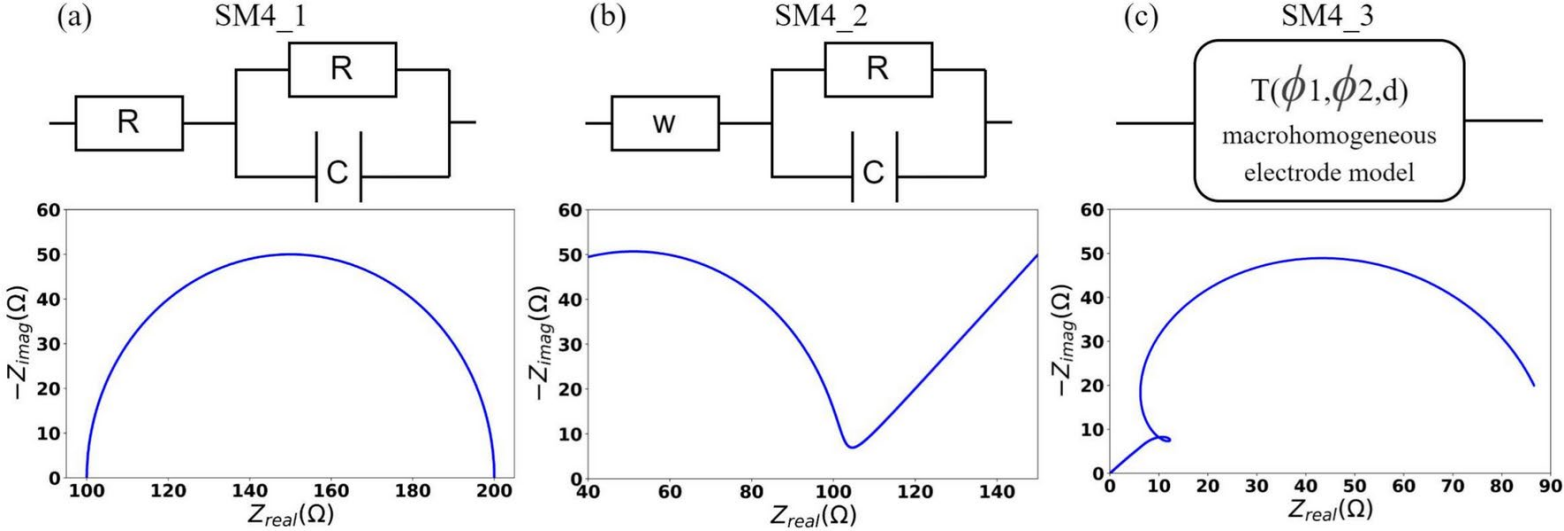
Circult diagrams and the corresponding nyquist plots of SM4_1, SM4_2, SM4_3.

The impedance.py package for analyzing electrochemical impedance spectroscopy (EIS) is employed to construct simulation models (SM) for data generation^21^. EIS involves applying a small amplitude sinusoidal alternating current voltage and measuring the corresponding AC current to obtain the impedance of an electrochemical system^22^. This package allows the construction of electrical circuits with different topologies and customisable circuit elements. Given the frequency of the applied voltage signal, the impedance.py enables the calculation of the corresponding impedance of the constructed electric circuit as feedback. In our DOE comparative studies, impedance.py was adopted to build SMs for data generation. The elements constituting the electric circuit and their value ranges, along with the frequency range of the voltage signal, form the input part of the parameter space. The magnitude of the impedance calculated by the impedance.py constitutes the output part of the parameter space. Six electric circuits of varying complexity were constructed for data generation. The details of these electric circuit SM are recorded in Table 1. The symbols for input parameters in Table 1 are referenced from a web source^23^. The number following each symbol indicates the quantity of that circuit component in the electric circuit. For example, SM4_1 includes two resistor components R1 and R2. Figure 3 presents the circults of SM4_1, SM4_2, and SM4_3^24^ and the corresponding Nyquist plots of some simulation models for a given frequency range.

**Table 1:** Codename, input parameter, and output parameter of the simulation models built with impedance simulation.

Codename	Input parameter
SM4_1	$\textrm{R}_1$, $\textrm{R}_2$, C, f
SM4_2	W, R, C, f
SM4_3	T(${\varphi }_1$,${\varphi }_2$,d), f
SM8_1	$\textrm{R}_1$, $\textrm{R}_2$, $\textrm{C}_1$, $\textrm{R}_3$, $\textrm{C}_2$, $\textrm{R}_4$,$\textrm{C}_3$, f
SM8_2	$\textrm{Gs}_1$, $\textrm{Gs}_2$, $\textrm{Gs}_3$, $\textrm{R}_1$, $\textrm{C}_1$,$\textrm{R}_2$, $\textrm{C}_2$, f
SM8_3	T(${\varphi }_1$,${\varphi }_2$,d), R, C,$\textrm{W}_1$, $\textrm{W}_2$, f

Following the definition of complexity in previous section, the complexity of these six SMs is quantified.

**Fig. 4:**
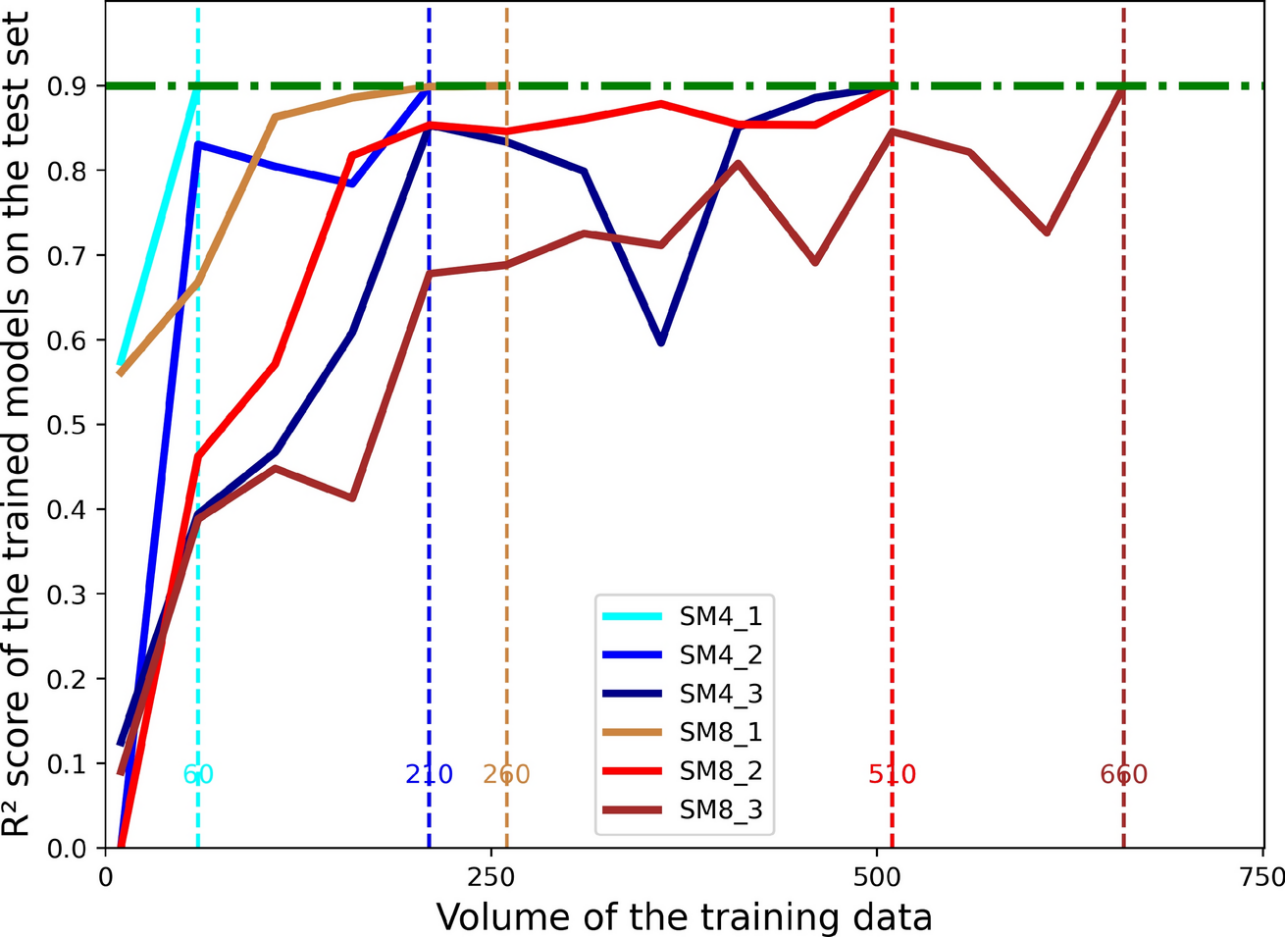
Complexity of the constructed SMs defined as the data volume required to train a surrogate predictive model to achieve a minimum performance of 0.9 R$^{2}$ score.

From Fig. 4, it can be observed that the complexity of SM4_3 and SM8_3 is significantly higher compared to other SMs, whereas SM4_1, SM4_2, and SM8_1 exhibit the lowest complexity.

### AL sampling strategies

AL sampling strategies adopt different model-based iterative sampling schemes within an iterative framework^25,26^. Starting from a set of initial data points, AL sampling iterates the following steps to generate new sample points in a given parameter space:

1. Fit a predictive model to the existing data;
2. Find the next point with the highest predictive uncertainty, Information gain, etc., according to the trained model;
3. Add this new data point to the existing dataset.

AL sampling can allocate data resources specifically to the relationships between input and output parameters in the parameter space, rather than following a predetermined distribution. However, the application of the iterative scheme is inherently limited. It needs an initial amount of data for subsequent iterations. Moreover, when the data acquisition process is time-consuming, the overall data acquisition investment for the iterative scheme is unacceptable in practice: determining the second new data point must be done after the first new data point is analyzed. Such limitation can be partially compensated for by the use of batch during iterations. The AL sampling strategies tested in this work includes: a Gaussian process-based AL sampling strategy using Emukit (Emu_GP)^27^, a Query by Committee (QBC) sampling strategy based on ModAL python package (ModAL_QBC)^28^, and a sampling strategy using Monte Carlo Dropout for uncertainty estimation implemented with the Baal python package (Baal_MCD)^29^. The latter two AL sampling strategies are self-reproduced algorithms based on information provided in relevant literature^30,31^. More details regarding the tested AL strategies are available in the supporting information.

#### Emu_GP

Emu_GP drives the AL sampling loop with a Gaussian process (GP) model. The advantage of GP is that it gives the predicted value while being able to return the uncertainty of the prediction measured in variance. The data point where the model yields the highest marginal predictive variance $\sigma ^2(x_*)$ is chosen as the new sample point.

2 $$\begin{aligned} \sigma ^2(x_*) = K(x_*, x_*) - K(x_*, X) K(X, X)^{-1} K(X, x_*) \end{aligned}$$

As illustrated in equation (2), $K(x_*, x_*)$ represents the covariance of a data point with itself for any potential new data point $x_*$, *K*(*X*, *X*) is the covariance matrix of the existed data points, $K(x_*, X)$ and $K(X, x_*)$ are the covariance vectors between the new point and the existed points^32^.

#### ModAL_QBC

For ModAL_QBC, its committee consists of about 30 regression models provided by scikit-learn^33^. These regression models $\{h_1, h_2, \dots , h_m\}$ provide an estimation towards prediction divergence and the position with the highest prediction divergence in the parameter space is then selected as the new sample point. As shown in equation (3), in which $P(h_i(x))$ refers to the prediction of model $h_i$.

3 $$\begin{aligned} \text {Prediction divergence}(x) = \frac{1}{m} \sum _{i=1}^{m} \left\| P(h_i(x)) - P_{\text {avg}}(x) \right\| ^2 \end{aligned}$$

#### Baal_MCD

Baal_MCD strategy employs a neural network as the core model, using a 50% dropout rate to randomly discard internal neurons to assess the divergence in predictions. In the same way as ModAL_QBC, the data point with the highest prediction divergence in the parameter space is chosen as the next sample.

### Study design

The data experiments were conducted according to the workflow described in “Methods”. a CCD, a LHD, and three AL sampling strategies were employed for this comparative study. Each selected DOE strategy guides the generation of 10 Datasets for a specific data volume within each parameter space. 10 AutoML modeling runs are conducted for each generated dataset. In the data generation process, a 50%-50% split between the initial data volume and the iterative data volume was uniformly adopted for all AL sampling strategies. During iteration, one single data point with the greatest information gain or uncertainty is selected. For the SM with 8 input parameters, the range of training data volumes is defined from 24 to 320 (data ratio 3-40). This means that only a very small amount of data is adopted for the modelling (12 training data points for the SM4_X series and 24 training data points for the SM8_X series). It is therefore understandable that the resulting models at the begining perform poorly on a test set containing 200,000 data points, especially when the parameter space complexity is high. For a given parameter space, the comparative study of DOE strategies are conducted at each specific data volume. As a statistical DOE strategy, the number of data points required by CCD is determined by the number factors and the design type, making it impossible to arbitrarily generate a CCD strategy for a specific data quantity. A D-optimal strategy^34^ which maximizes the determinant of the information matrix is employed to select subsets of CCD for a given data quantity. These subsets can then be compared with other DOE strategies at the same data volume.

### Implementation of the DOE comparative workflow

The simulations in this paper are performed on a high performance computing (HPC) platform. Table 2 provides information about the hardware settings of this HPC platform. More details are provided in our GitHub repository.

**Table 2:** Hardware settings of the HPC platform.

	Value
Architecture	x86_64
CPU type	Intel(R) Core(TM) i5-9500
Core(s) per node	6
Base clock speed	3.00 GHz
Number of nodes	20

## Results

### Observations on noise-free data

**Fig. 5:**
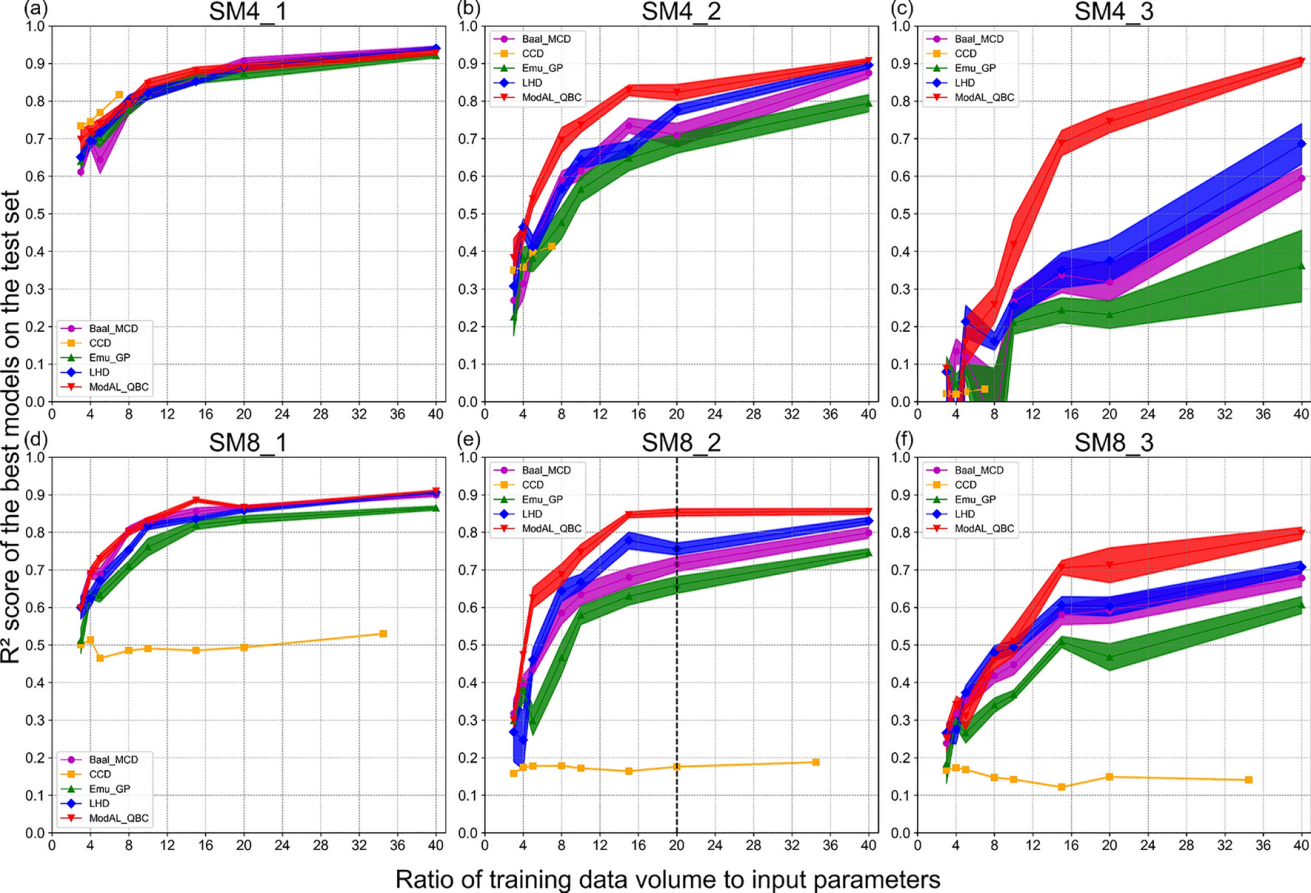
Results of DOE performance for varying DOE strategies, noise levels and model complexities w.r.t. ratio of training data to input parameters.

Results of the DOE comparative analysis are recorded in Fig. 5, the vertical axis of each subplot represents the R$^{2}$ score of the AutoML-trained models on the test set. The horizontal axis shows the ratio of data volume to the dimensionality of the input parameter space.

The stochasticity of the DOE strategies and the confidence intervals of model evaluations are indicated by shaded areas of the same color in Fig. 5. As a predefined DOE strategy, CCD does not have stochasticity during data allocation. Therefore, its confidence interval is solely comprised of the uncertainty in model evaluation. The low-transparency shading area reflects the stochasticity of the DOE strategies, which is quantified by generating 10 datasets at each given data volume. The high-transparency shading area reflects the uncertainty in model evaluations. When comparing two DOE strategies at a specific data volume, a reliable distinction (95% confidence level) can be made only if their confidence intervals do not overlap. For instance, in the parameter space defined for SM4_3, the ModAL_QBC strategy exhibits a statistically significant superiority over the other strategies once the data volume exceeds eight times the dimensionality of the input parameters. Conversely, the differences among the Baal_MCD, Emu, and LHD strategies are not distinctly discernible. In all parameter spaces, narrower confidence intervals for any DOE strategy can be observed as the amount of training data increases, i.e., the stochasticity of the DOE strategy is reduced with increasing data volume. To reduce the impact of stochasticity associated with DOE strategies, more datasets need to be generated and modeled with AutoML to narrow the scope of DOE stochastic sampling.

### Observations on noisy data

The comparative experiments of DOE strategies on imprecise datasets are carried out following the workflow described in “Methods” section. During DOE-guided data generation, a noise characterized by a uniform distribution was added to the target parameters, as illustrated by the equation below.

4 $$\begin{aligned} y\_noisy= y + Z \end{aligned}$$

Z follows a uniform distribution $Z \sim U(-\alpha , \alpha )$ characterised by $\alpha$. $\alpha$ is related to the range of values of the target parameter in the given parameter space. Gaussian noise has a greater likelihood of generating low level noise points near zero. These noise points are not representative of the set level of uncertainty. As a result, uniformly distributed dominated noise is chosen over Gaussian noise. For a given noise level, ten datasets with noise contenmination are generated for each tested DOE strategy. With different random seeds, the specific noise magnitude on each target parameter varies across these datasets. Similarly, each dataset is subjected to ten training sessions to obtain an approximated optimal prediction model.

**Fig. 6:**
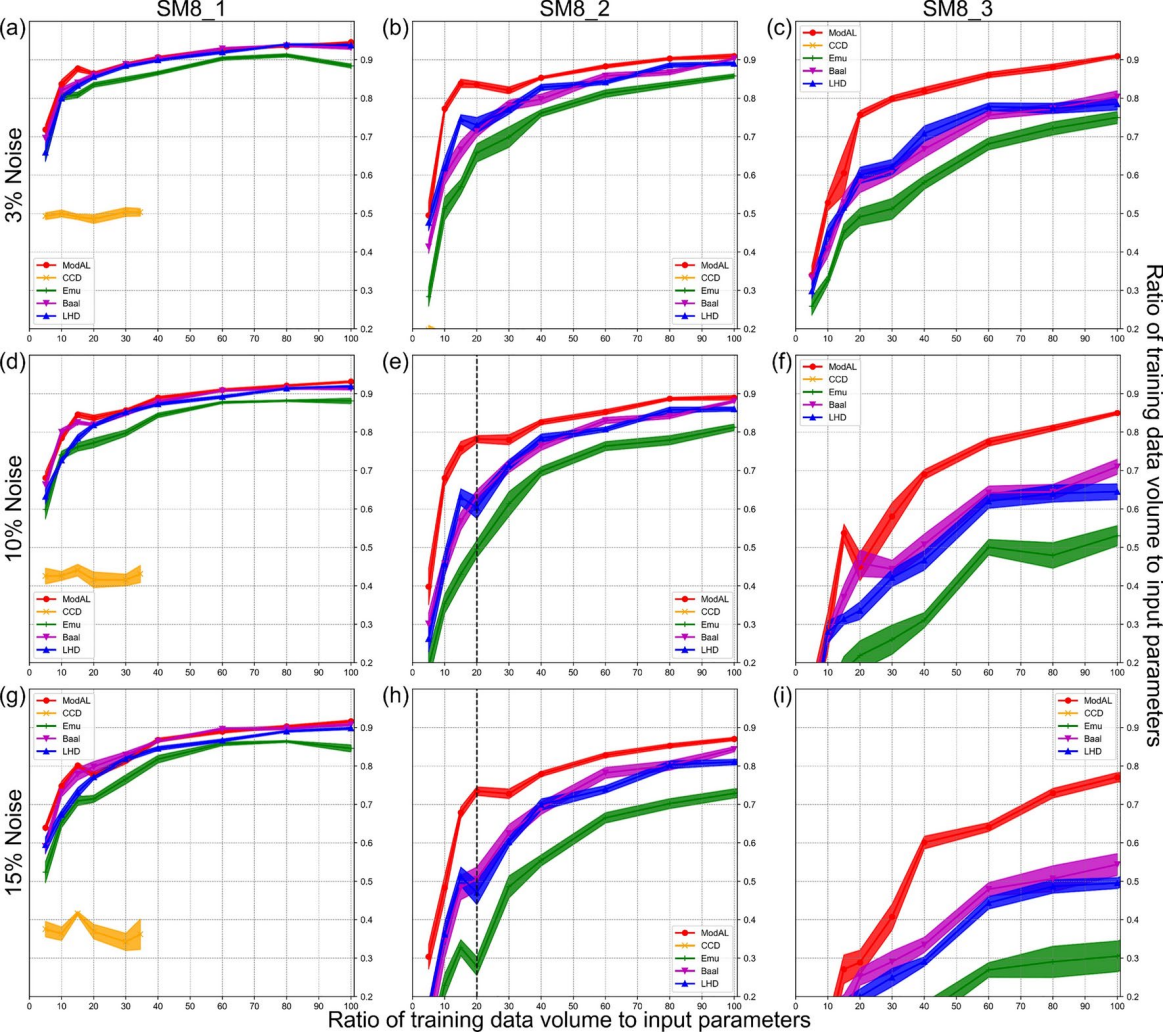
Results of a DOE comparison study with varying noise levels( $\alpha$ = 3%, 10%, 15%).

The comparative experiments of DOE strategies are divided into two parts. The first part of the comparative experiments are conducted in parameter space defined by SM8_1, SM8_2, and SM8_3, aiming to explore the impact of noise on different DOE strategies. The noise level $\alpha$ is set in relation to the value range of the target parameter with three levels: 3%, 10%, and 15%. LHD strategy with a fixed random seed, ModAL_QBC strategy, and Emu_GP are selected as experiment candidates. The results of these comparative experiments are summarized in Fig. 6.

The second part of the comparative experiments focuses on the applicability of replications in data allocation. Confronted with the presence of noise contamination in the data, classical DOE obtains information regarding uncertainty through repetitions of experiments (replication) to examine whether the assumptions of using a certain statistical tool are met. This approach aims to enhance the precision and reliability of experimental results. However, in the realm of ML-driven data analysis, the concept of replication is nearly abandoned. On one hand, the presence of big data, or more precisely adequate sample points, inherently possesses noise-reducing effect to some extent by averaging data points located close to each other. This makes it no longer cost-effective to repeat experiments entirely on known positions: replications fail to yield new information. On the other hand, data cleaning approaches employed during data preprocessing can effectively reduce the adverse impacts of noise. These two advantages of ML render the adoption of replications in data planning less economical. Replication only become worthwhile when the effective noise level (0.5 $\alpha$ in case of uniform distributed noise) surpasses a threshold where the improvements in model performance, achieved by noise reduction in the training data, compensate for the reduction of available data resources for parameter space sampling. Simulation experiments were carried out in the parameter space delineated by SM8_1 and SM8_2 to compare the suitability of the four strategies: LHD, LHD performing one replication (LHD_r1), with half of the data for exploration and the other half for repeated experiments, LHD performing three replications (LHD_r3) and LHD performing seven replications (LHD_r7). Specifically, datasets constructed using replication strategies offer two possibilities for guiding the generation of predictive models:

- Obtaining a dataset that is half (a quarter for LHD_r3) the original size by averaging the values of the target parameter, and using this noise-reduced dataset as input data for AutoML modeling
- Dataset that includes repeated experiments are directly fed as input data for AutoML modeling.

**Fig. 7:**
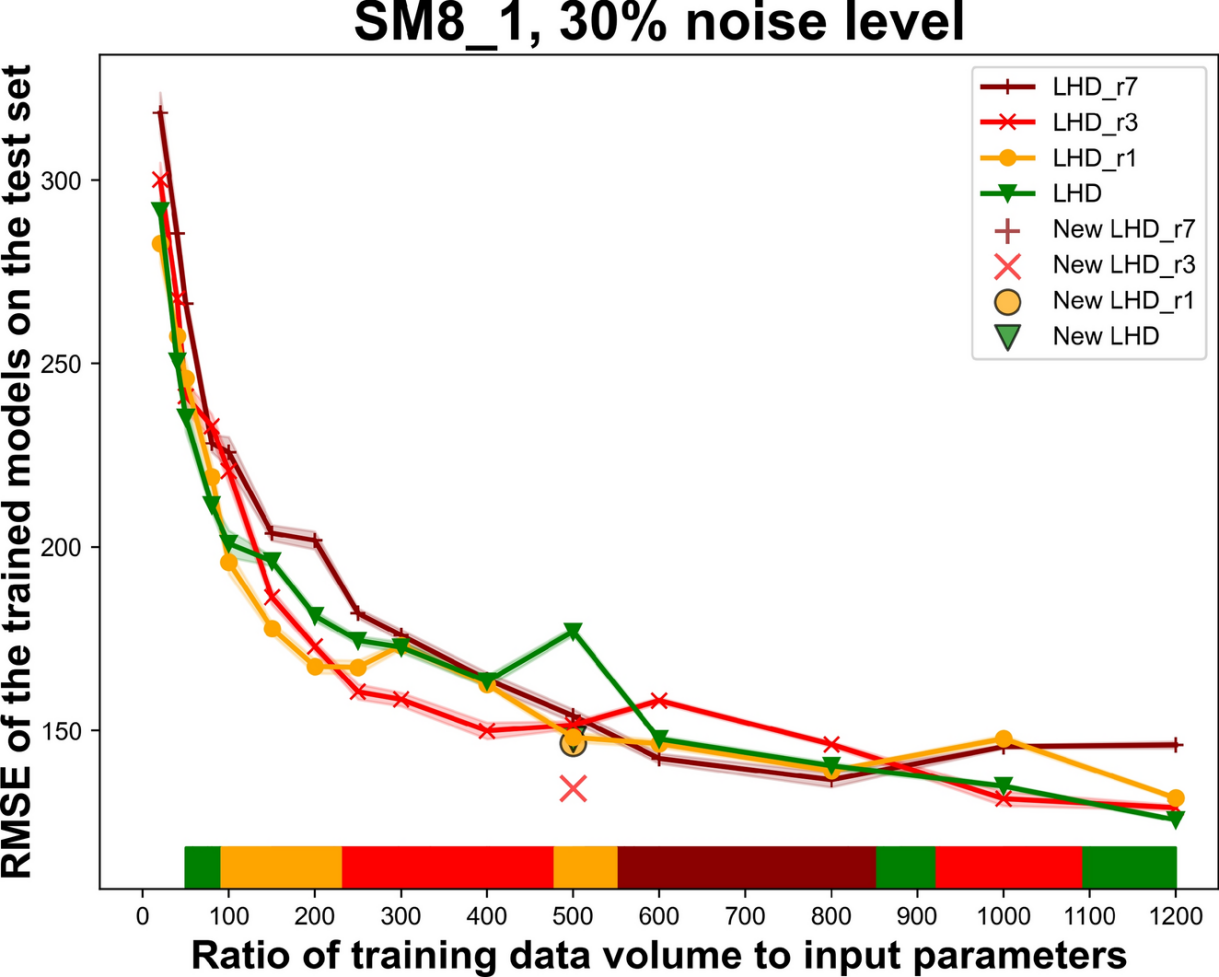
Replication in data distribution versus only sampling with new data points, the color bar illustrates the best performing DOE strategy, an extra set of simulation experiments was performed at ratio=500 with longer AutoML training duration.

The former was chosen for model training in this comparative study. The complete experimental results are presented in supplementary material. Figure 7 illustrates the performance of LHD and its replication variants in parameter space SM8_1 with $\alpha$ = 30% across data ratio 50 to 1200, using the Root Mean Squared Error (RMSE) of the prediction model as the metric. The color bar on the x-axis indicate the best-performing strategy at different data quantities. Due to the instability of the AutoML package on the HPC platform, the model trained with LHD at ratio 500 has an abnormal RMSE increase. In this regard, an extra set of simulation experiments was performed at ratio=500 with longer AutoML training duration (300s instead of 200s). It is worth noting that both sets of results support the discussion and conclusions about replication.

## Discussion

The choice of DOE strategy is discussed in this section based on the results of the simulation experiments. Attention is drawn to the role of complexity and noise in DOE selection. Furthermore, the adoption of noise reduction approaches, i.e. data for replications^35^ versus data for parameter space exploration are fully discussed.

### Impact of complexity on the data allocation capability of DOE strategies

By comparing subplots (a), (b) and (c) or (d), (e), and (f) in Fig. 5, it can be observed that the stochasticity of the DOE strategy is related to the complexity of the parameter space. As the complexity increases the stochasticity of each DOE strategy rises accordingly. As a classical statistical DOE strategy, CCD performs best in a simple parameter space with 4 dimensions (SM4_1). However, in higher-dimensional (8D) spaces or in complex low-dimensional parameter spaces (SM4_2, SM4_3), CCD lacks adequate data distribution capabilities.

The AL sampling strategies are not always superior to conventional DOE strategies as reported in some studies. As shown in Fig. 4, the performance of LHD and Baal_MCD strategies cannot be distinguished in most parameter spaces. In parameter spaces of medium complexity (e.g. SM4_2, SM8_2), the LHD strategy even outperforms Baal_MCD at most data volumes. The ModAL_QBC strategy demonstrates superior data allocation capability over LHD in all parameter spaces when sufficient data volume is sufficient. The differences between ModAL_QBC and LHD become more pronounced as the complexity of the interrelationship between input parameters and target parameters increases. However, the advantages of the AL sampling strategy appear only when the data volume is sufficient. The sufficiency of the data volume is related to the complexity of the parameter space defined. Comparing the results of SM4_2 and SM4_3 or SM8_2 and SM8_3, it can be seen that as the complexity of the parameter space increases, ModAL_QBC reveals its superiority over LHD only with a higher amount of data (higher ratio). Increasing the proportion of the initial dataset in cases of small data volume can improve the performance of AL sampling strategies.

### Impact of noise on the data allocation capability of DOE strategies.

Figure 6 documents the performance of the five DOE strategies in the presence of noise contamination. CCD is extremely poorly behaved in complex parameter space such as SM8_2 and SM8_3, meaning it cannot provide any meaningful data allocation. Its performance cannot be shown in the interval with R$^{2}$ score higher than 0.2. Therefore the CCD’s performance cannot be shown in some subplots in Fig. 6. The performance of all trained models declined after adding noise to the training dataset. The higher the noise level, the more significant the performance degradation compared to the noise-free condition. To achieve a performance level similar to that of the noise-free condition, a larger training dataset is required. Table 3 shows the total amount of data required to obtain a predictive model reaches an R$^{2}$ score of 0.8 in different parameter spaces at different noise levels. As can be seen from Table 3, for SM8_3, which has high complexity, an additional 3 times the amount of data is required to overcome the 10% level of noise to make the trained model reach R$^{2}$=0.8.

**Table 3:** Factor of original data volume required to obtain a predictive model with an R$^{2}$ = 0.8 at different noise levels.

	3% Noise	10% Noise	15% Noise
SM8_1	1.07	2	2.4
SM8_2	1.43	3	4
SM8_3	2.14	4	7

DOE strategists show varying sensitivities to noise impacts. Table 4 records the performance of four DOE strategies at data ratio=20 in parameter space defined by SM8_2 (labelled with vertical dashed lines in Fig. 5 and 6). As shown in Table 4 , among the four evaluated DOE strategies, ModAL_QBC demonstrates the best robustness to noise, while Emu_GP is the most susceptible. Its performance at each data volume is significantly weaker than that of ModAL_QBC or LHD at the same noise level. As the noise level increases, this gap becomes more noticeable. Based on the results of the simulation experiments, the AL committee strategy (ModAL_QBC) exhibits excellent noise resistance when the target parameters are contaminated. When performing DOE selection to learn from noisy data in practice, it is recommended to conduct a prestudy to assess the noise-handling capabilities of the candidate DOE strategies.

**Table 4:** Performances of Emu_GP, ModAL_QBC, Baal_MCD, and LHD strategy measured by R$^{2}$ score at ratio 20 (training data volume to input parameters, 160 training data) in parameter space SM8_2.

	Emu_GP	ModAL_QBC	Baal_MCD	LHD
0% noise	0.66	0.85	0.72	0.76
10% noise	0.49	0.78	0.63	0.61
15% noise	0.28	0.73	0.51	0.48

### Role of replication for noise suppression

This article does not employ uncertainty quantification to assess the applicability of replications mathematically. Instead, it aims to provide guidance for considering the applicability of replications from a practical perspective. From Fig. 7, it can be observed that replications are suitable for a moderate range of data volumes (ratio 100 - 1000). As the overall noise level decreases, the advantage of multiple replications diminishes. Single replication initially demonstrates its advantage within the applicable range of replications. As the data volume increases, using multiple replications becomes beneficial. This phenomenon is not only observed in the SM8_1. Replication is not the optimal choice in small data scenario or big data. In small data scenarios, exploring the parameter space for more information is clearly more efficient. With big data, on the other hand, replication can be replaced through the noise-reducing effect brought by intense sampling. Additionally, as the noise level increases, the applicable range of the replication strategy expands. The noise reduction effect of replications can be calculated with equation (5). For example, performing 3 replications can reduce the effective noise impact to half of its original level. It is important to note that the discussed noise reduction effect pertains to the effective noise impact.

5 $$\begin{aligned} \text {statistically reduced noise (training data)} = \frac{ effective \ noise \ impact \ characterized \ by \ \alpha }{\sqrt{times \ of \ replication + 1}} \end{aligned}$$

The core criterion for determining the applicability of replication is to compare the noise reduction effect achieved by the portion of data used for replication and its resultant improvement in model performance with the improvement brought about by directly sampling this portion of data in the parameter space. From the results of the conducted simulation experiments (documented in the supplementary material), it can be deduced that when the RMSE desrease of the model performance provided by using the data directly for further sampling is higher than one-fifth of the noise reduction in the training data, it is advantageous to use the data points exclusively for sampling. Only for intermediate data volume ratios (100-1000) it becomes profitable to start considering the use of replication to directly reduce noise contamination in data acquisition. Nevertheless, this rule of thumb requires the user to being able to estimate the model performance boost (RMSE reduction) that a given amount of data can support.

## Application of the proposed AutoML-workflow for DOE selection

It is worth reiterating that our recommendations for DOE selection mainly address the objective of incorporating machine learning to obtain a mathematical model in order to predict further response. In practice, selecting a DOE and then conducting experiments or production can be considered a one-time event. An unreasonable or even incorrect choice of DOE may at best lead to a suboptimal solution, and at worst, result in the failure of data analysis. Thus, a simulated environment that closely resembles real-world conditions offers an almost zero-cost method for the testing of possible DOE solutions, serving as a valuable reference for actually making DOE choice.

The AutoML-based workflow proposed in this paper provides such a framework for evaluating and selecting DOE strategies. Once the available data volume and the number of factors to be included in subsequent data analysis are determined, the complexity of the parameter space can be estimated based on physical knowledge or user experience. A SM resembling can then be constructed as a playground that simulates real-world conditions for DOE testing. The noise level in the data should also be considered. with sufficient data resources, operators can conduct multiple repeated experiments during the pre-study or pre-production phase to obtain the variability of the target parameter. In cases where data is limited, the noise level can be estimated through indirect methods, such as considering multiple sources of uncertainty and performing uncertainty propagation to estimate the combined uncertainty of the target parameter^36^.

Such simulated environment is particularly meaningful for the application of AL DOE strategies. AL often requires user expertise for case-specific hyperparameter optimization. The simulated environment facilitates the adaptive optimization of AL strategies for varying parameter spaces, which allows maximizing the effectiveness of AL in data allocation while reducing the risk of incorrect deployment. With references provided by simulation results, DOE candidates can be assessed w.r.t. the training data volume in a manner that close to real-world applications.

## Conclusion

To make machine learning based analysis fruitful in experimental investgations, a proper DOE strategy for dataset generation is required. This paper provides references and suggestions for choosing suitable DOE strategies in the case of noise contaminated and noise free data. To benchmark different DOE strategies, including various active learning sampling strategies, a workflow based on AutoML multi-modeling has been proposed: Each dataset generated by a DOE strategy is used for model creation within an AutoML framework to obtain the best predictive model achievable within a given model class. Hence, the performance of the best model indicates the data allocation capability of the corresponding DOE strategy. Throughout this workflow, the stochasticity inherent in DOE strategies, the uncertainties in model evaluation and noise in target parameters are also considered.

From simulation experiments with noise-free data, it is observed that not all AL sampling strategies outperform conventional DOE strategies, contrary to sometimes raised naive expectations. As a committee-based AL strategy, ModAL_QBC demonstrates superior performance and strong robustness against noise across parameter spaces of varying complexities. This superiority is particularly evident in highly complex parameter spaces. On the contray, Emu_GP is particularly susceptible to noise and therefore unable to allocate data effectively in case of noise contanmination.

From our observations we conclude that any DOE selection is best grounded on the determination of the possibly available data volume, the number of factors included in later data analysis, and the noise level in the data. With references provided by simulation models, suitable DOE choices are discussed w.r.t. the ratio of training data volume to input parameters.

In addition, this paper considers the application of identical data point replications in DOE planning. The comparison between parameter space exploration and repeated experiments is conducted -to our knowledge for the first time- with LHD and its variants for different replication factors. Our observations indicate that using all available data resources for parameter space exploration prevails until the effective noise level reaches 5% of the total range of the target parameter. This trade-off between parameter space exploration and replication is quantized with a rule of thumb derived from simulation results.

## Supplementary Information

Supplementary Information.

## Data Availability

The datasets generated and analysed during the current study are available in the repository https://github.com/xinchengxxc/AutoML-for-DOE-selection-and-reference-studies.

## Code availability

The code involved in this work is available on GitHub: https://github.com/xinchengxxc/AutoML-for-DOE-selection-and-reference-studies.
