# Supplementary material for: AutoML based workflow for design of experiments (DOE) selection and benchmarking data acquisition strategies with simulation models
Source: Sci Rep. 2024 Dec 31;14:32170. doi: 10.1038/s41598-024-83581-3 (PMC11688508; doi:10.1038/s41598-024-83581-3)
Supplement: Supplementary file 1 — Supplementary Information. [file 41598_2024_83581_MOESM1_ESM.docx]

Supplementary Material


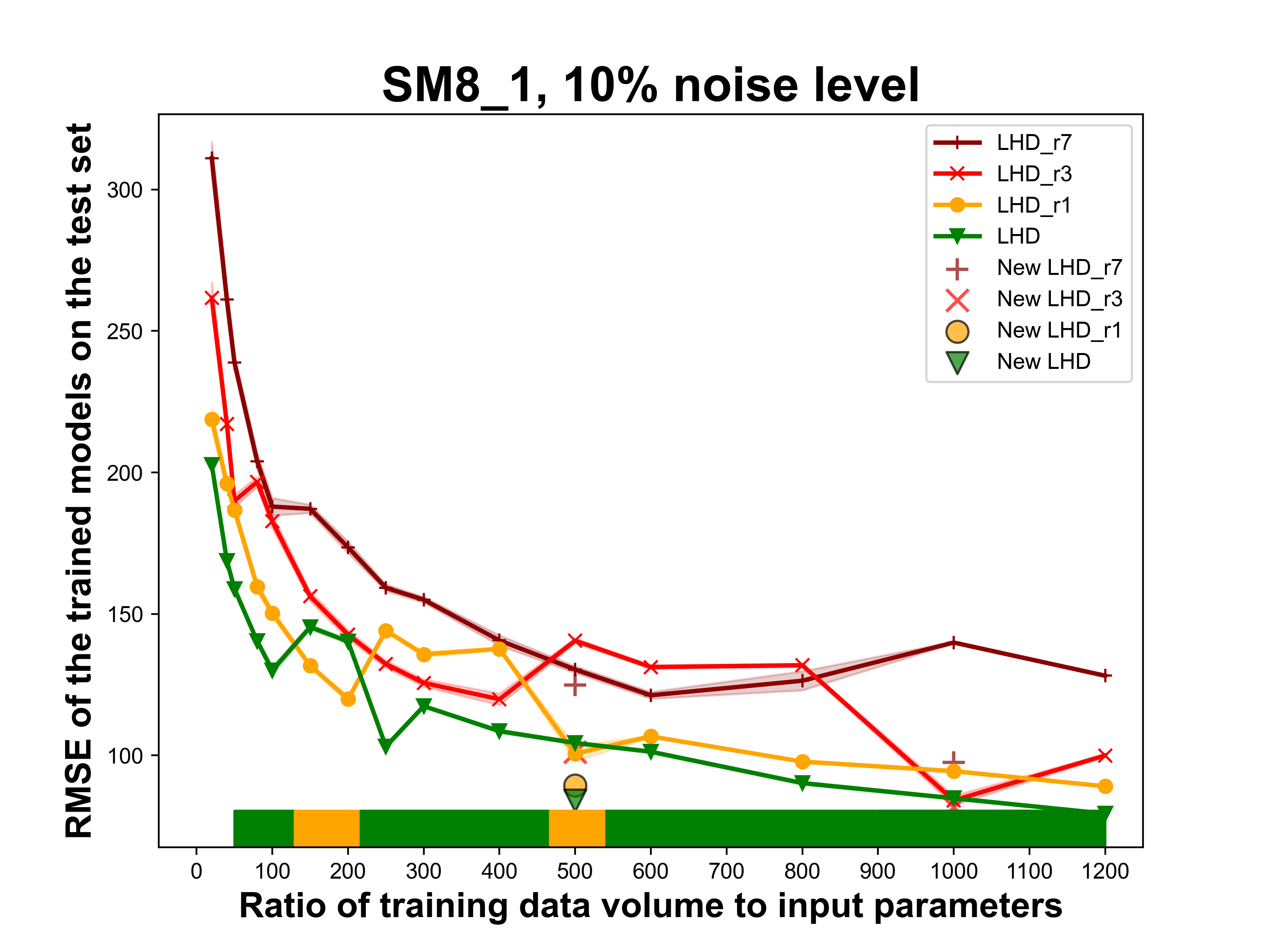


Supplementary Figure 1. Replication in data distribution versus only sampling with new data points, SM8_1, 10% noise level. The color bar illustrates the best performing DOE strategy.


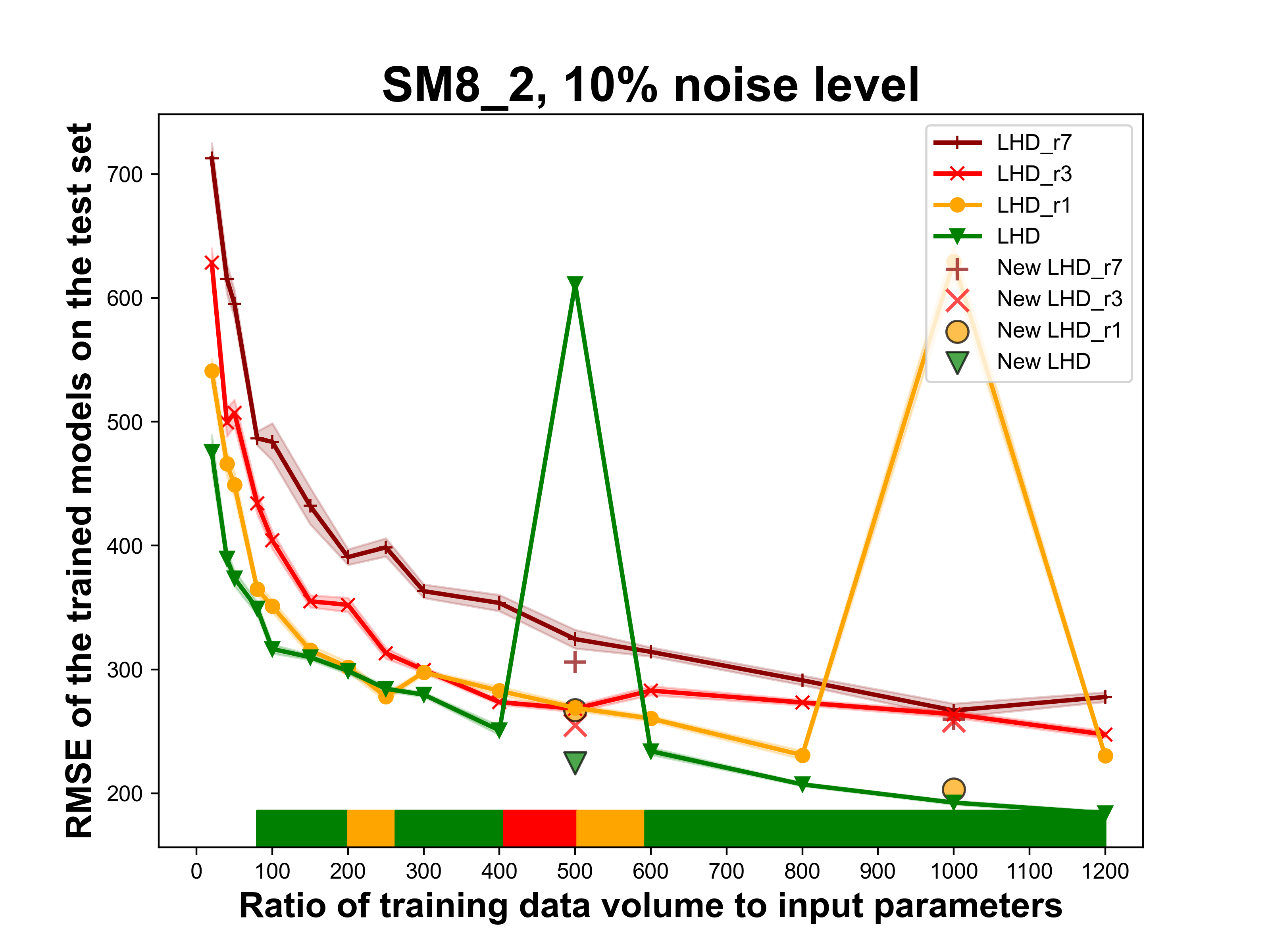


Supplementary Figure 2. Replication in data distribution versus only sampling with new data points, SM8_2, 10% noise level. The color bar illustrates the best performing DOE strategy.


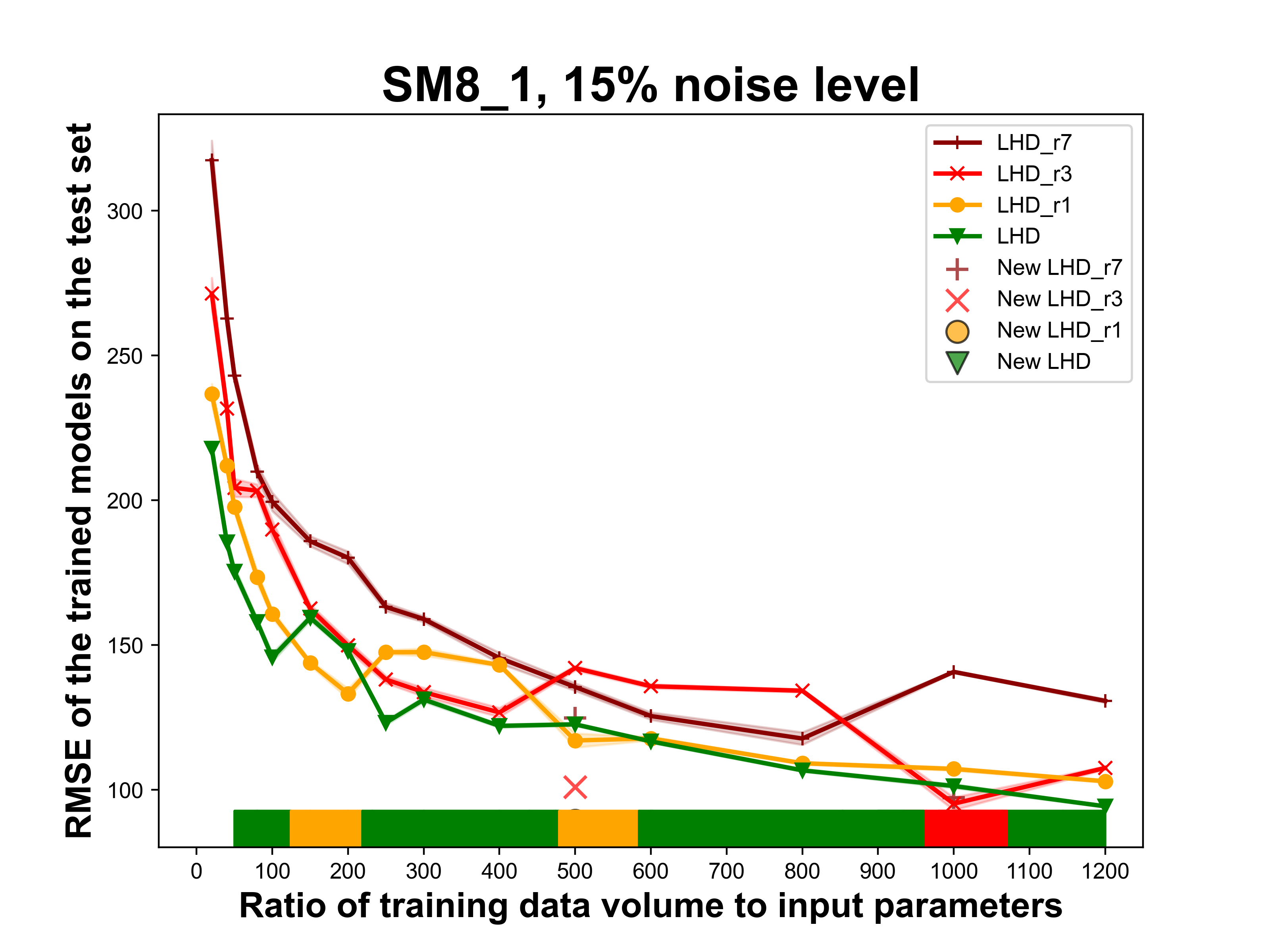


Supplementary Figure 3. Replication in data distribution versus only sampling with new data points, SM8_1, 15% noise level. The color bar illustrates the best performing DOE strategy.


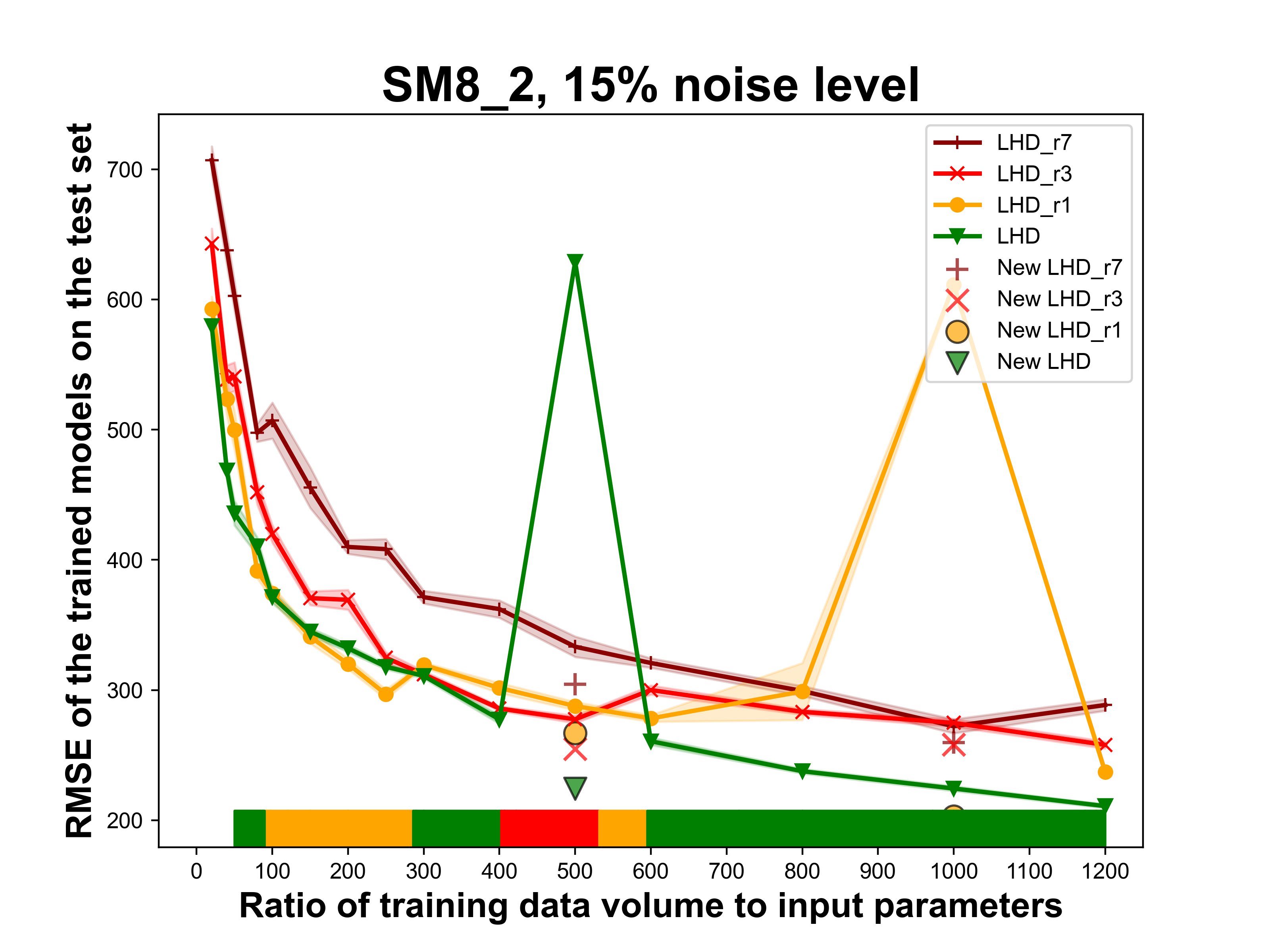


Supplementary Figure 4. Replication in data distribution versus only sampling with new data points, SM8_2, 15% noise level. The color bar illustrates the best performing DOE strategy.


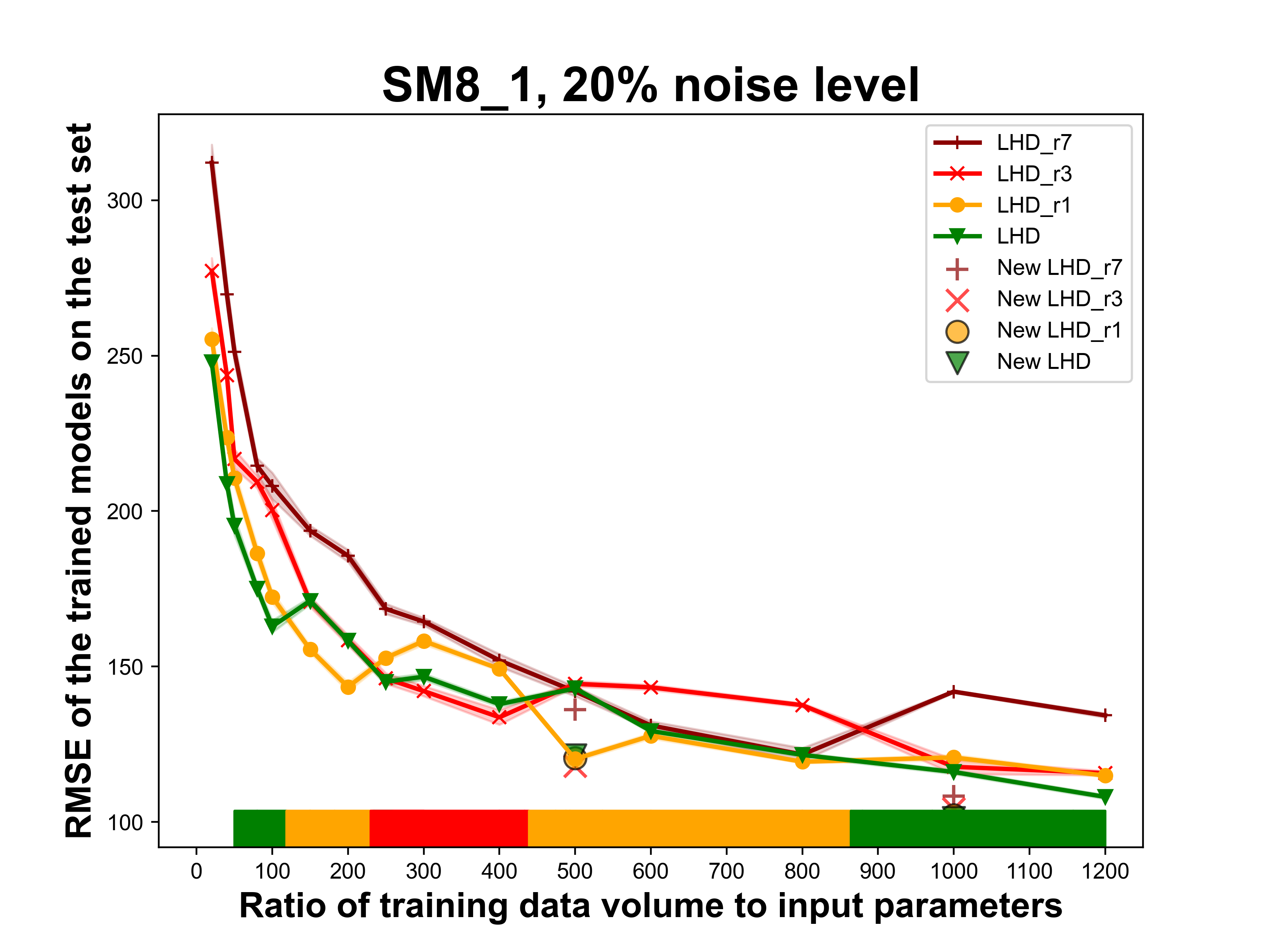


Supplementary Figure 5. Replication in data distribution versus only sampling with new data points, SM8_1, 20% noise level. The color bar illustrates the best performing DOE strategy.


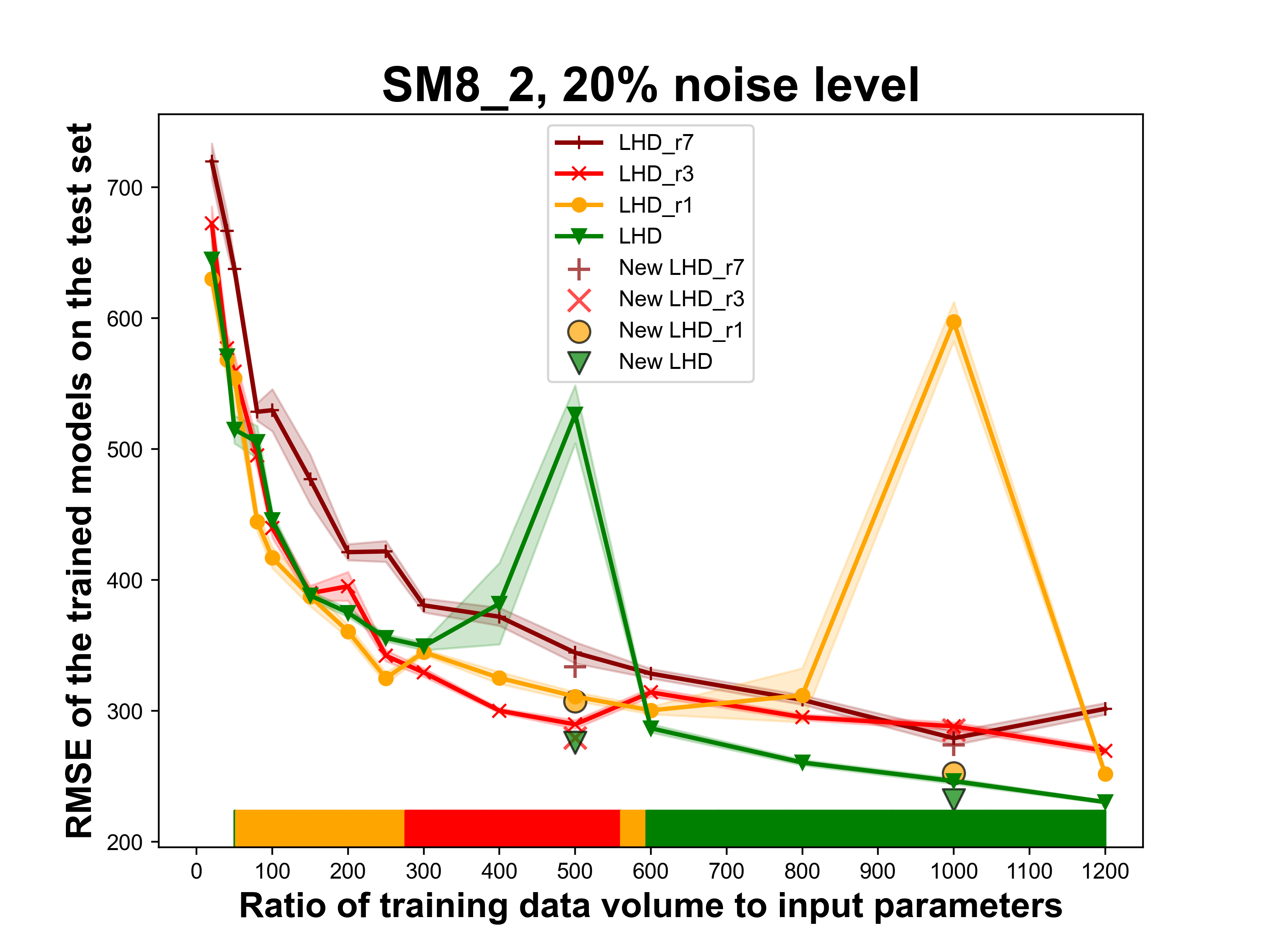


Supplementary Figure 6. Replication in data distribution versus only sampling with new data points, SM8_2, 20% noise level. The color bar illustrates the best performing DOE strategy.


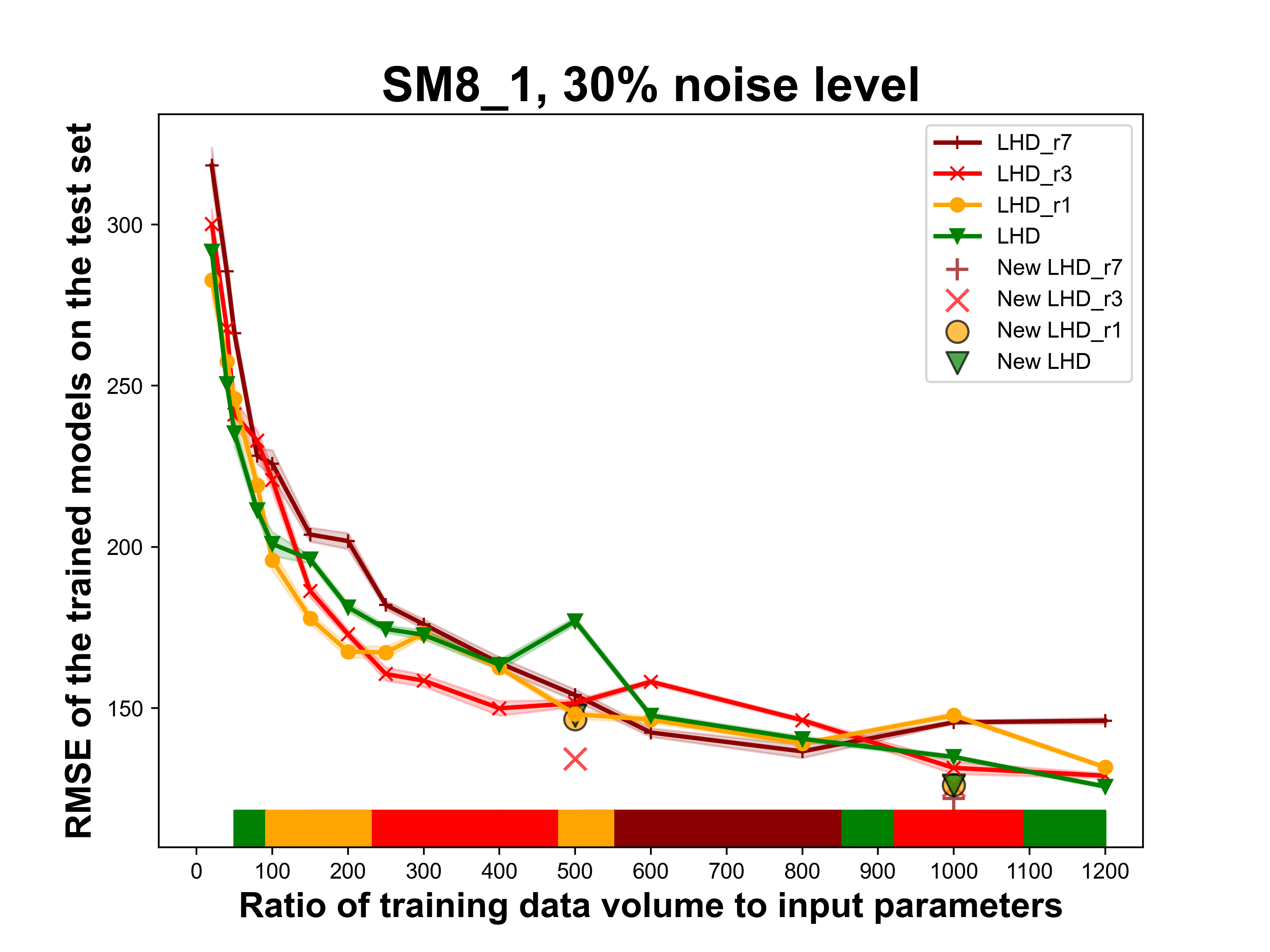


Supplementary Figure 7. Replication in data distribution versus only sampling with new data points, SM8_1, 30% noise level. The color bar illustrates the best performing DOE strategy.


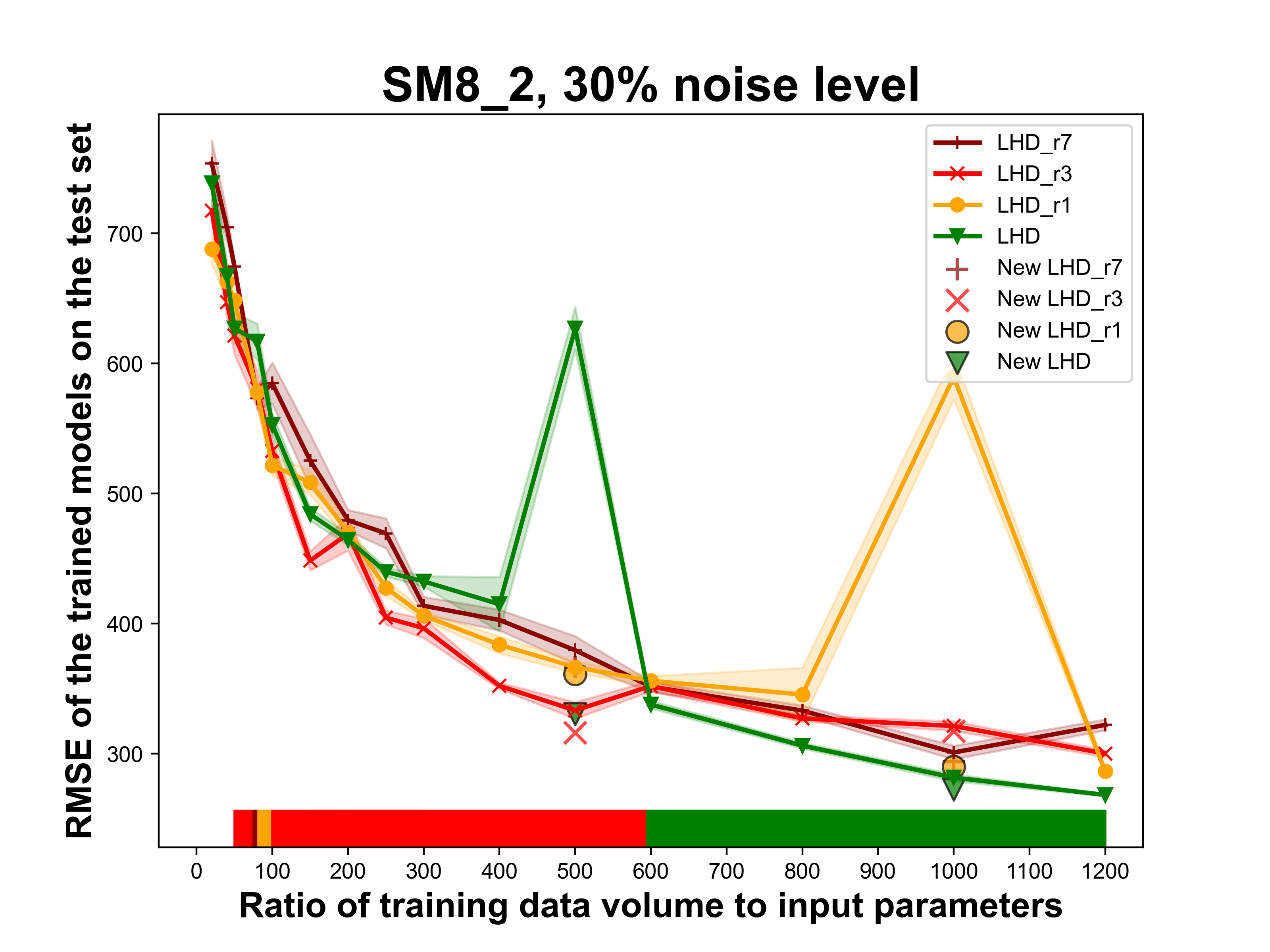


Supplementary Figure 8. Replication in data distribution versus only sampling with new data points, SM8_2, 30% noise level. The color bar illustrates the best performing DOE strategy.

Details about the AL strategies tested in this work

ModAL_QBC:

Iteration ratio: 50% data points as initial data, 50% data points for AL iteration;

Members (models) in the Committee ($v$ represents variable):

RandomForestRegressor (n_estimators = $v$, min_samples_split = $v$);

AdaBoostRegressor (n_estimators = $v$);

LinearRegression ();

BayesianRidge ();

MLPRegressor (hidden_layer_sizes = $v$, activation = $v$, batch_size = $v$, max_iter=5000);

Uncertainty acquisition: standard deviation, choose the next data point at the location where the predictions of the models has the highest standard deviation;

Baal_MCD:

Iteration ratio: 50% data points as initial data, 50% data points for AL iteration.

Neural network: layers constructed with tensorflow in python,

nn.Sequential(nn.Linear(dimension, dimension*4), nn.ReLU(), nn.Linear(dimension*4, dimension*3), nn.ReLU(),

nn.Linear(dimension*3, dimension*2), nn.ReLU(), nn.Linear(dimension*2, dimension), nn.ReLU(), nn.Linear(dimension, 1);

Loss function: mean square error;

Optimizer = optim.Adam(model.parameters(), lr=0.005);

Hyperparameters: n_epochs = 3000, batch_size = 30;

Layer drop out: 50%

Uncertainty acquisition: mean square error, choose the next data point at the location where the predictions of the neural networks (with different drop-outs) has the highest mean square error;

Emu_GP:

Iteration ratio: 50% data points as initial data, 50% data points for AL iteration;

Kernel: GPy.kern.Bias(input_dim=dimension) + GPy.kern.Bias(1.0) * GPy.kern.RBF(input_dim=dimension, variance=1., lengthscale=1.,ARD=True) + GPy.kern.Bias(1.0) * GPy.kern.Matern32(input_dim=dimension, variance=1., lengthscale=1.);

Uncertainty acquisition : Choose the next data point at the location where the model for predicting target parameter $f(x)$ has the highest marginal predictive variance $a_{US}\left( x \right)=\sigma^{2}(x)$;

Optimizer: gradient acquisition optimizer;

The error between model predictions and the true value of the target parameter within test data set, tested with 2 randomly selected models.

| 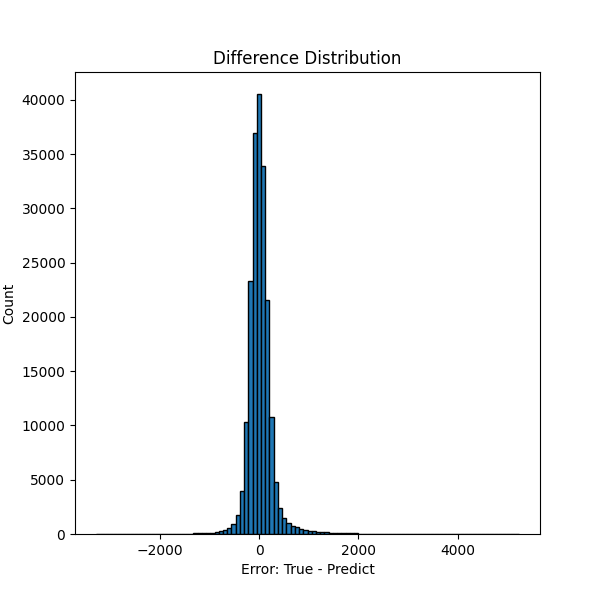 | 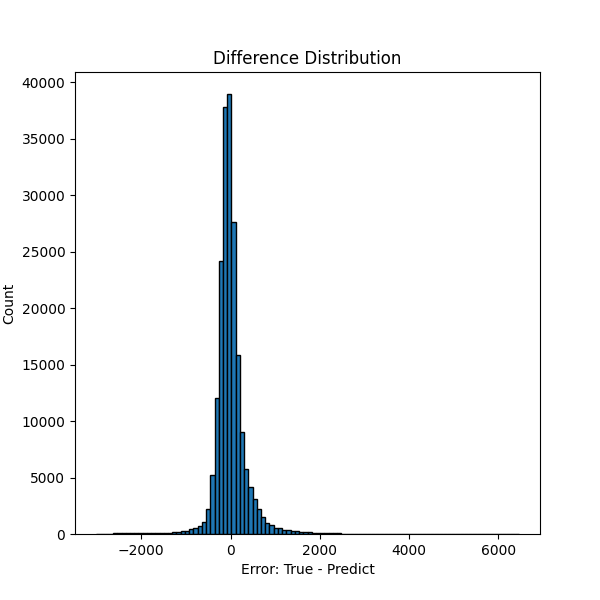 |
| --- | --- |
